# Visually estimated ejection fraction by two dimensional and triplane echocardiography is closely correlated with quantitative ejection fraction by real-time three dimensional echocardiography

**DOI:** 10.1186/1476-7120-7-41

**Published:** 2009-08-25

**Authors:** Kambiz Shahgaldi, Petri Gudmundsson, Aristomenis Manouras, Lars-Åke Brodin, Reidar Winter

**Affiliations:** 1 Department of Cardiology, Karolinska University Hospital Huddinge, Stockholm, Sweden; 2 Department of Biomedical Laboratory Science, Malmö University, Malmö, Sweden; 3 Department of Clinical Physiology, Karolinska University Hospital Huddinge, Stockholm, Sweden; 4 School of Technology and Health, Royal Institute of Technology, Huddinge, Sweden

## Abstract

**Background:**

Visual assessment of left ventricular ejection fraction (LVEF) is often used in clinical routine despite general recommendations to use quantitative biplane Simpsons (BPS) measurements. Even thou quantitative methods are well validated and from many reasons preferable, the feasibility of visual assessment (eyeballing) is superior. There is to date only sparse data comparing visual EF assessment in comparison to quantitative methods available. The aim of this study was to compare visual EF assessment by two-dimensional echocardiography (2DE) and triplane echocardiography (TPE) using quantitative real-time three-dimensional echocardiography (RT3DE) as the reference method.

**Methods:**

Thirty patients were enrolled in the study. Eyeballing EF was assessed using apical 4-and 2 chamber views and TP mode by two experienced readers blinded to all clinical data. The measurements were compared to quantitative RT3DE.

**Results:**

There were an excellent correlation between eyeballing EF by 2D and TP vs 3DE (r = 0.91 and 0.95 respectively) without any significant bias (-0.5 ± 3.7% and -0.2 ± 2.9% respectively). Intraobserver variability was 3.8% for eyeballing 2DE, 3.2% for eyeballing TP and 2.3% for quantitative 3D-EF. Interobserver variability was 7.5% for eyeballing 2D and 8.4% for eyeballing TP.

**Conclusion:**

Visual estimation of LVEF both using 2D and TP by an experienced reader correlates well with quantitative EF determined by RT3DE. There is an apparent trend towards a smaller variability using TP in comparison to 2D, this was however not statistically significant.

## Background

Echocardiography is a noninvasive imaging technique that provides immediate assessment of global and regional LV function. LVEF is the most commonly used parameter of LV systolic function. The assessment of LV function is an important clinical tool and is of great clinical importance in the evaluation and prognosis of patients with coronary artery disease, hypertension, diabetes, valvular disease, and congestive heart failure [1]. The reliable assessment of LV systolic function is important for therapeutic decision making in heart failure [2].

EF can be assessed using a variety of modalities including contrast ventriculography [3], magnetic resonance imaging (MRI) [4] and echocardiography [5]. By far echocardiography is the least expensive, most widely available technique and it is noninvasive, can be performed bedside, is rapid and very safe. LVEF is a measure of LV systolic function that has been shown to predict mortality in a number of conditions [6-8].

However, 2DE has a number of well-known limitations, the two most important perhaps being LV foreshortening and that different projection are not possible to acquire during the same cardiac cycle. The latter is particularly important in patients with variable heart rhythm (i.e. atrial fibrillation). Both these limitations can be overcome using a 3D-array transducer for acquiring the apical four-two-and three chamber views simultaneously in the TP mode.

RT3DE has shown excellent agreement to MRI in measuring both LV volumes and EF and has also proven to be superior to quantitative 2DE regarding EF [9-11]. These data are suggesting that RT3DE is interchangeable with MRI in measuring LVEF.

Quantitative 2D-EF using BPS rule can be somewhat time consuming, and the endocardial border tracing is sometimes difficult to perform, especially in patients with poor image quality, since it is performed on still frames. In clinical practice eyeballing for the evaluation of LVEF can be done faster and is often easier to perform, especially in patients with poor image quality.

Previous studies have demonstrated the value of eyeballing EF [12-16] and one study revealed good correlation with quantitative 2DE measurements [12]. However, the studies comparing to radionuclide methods were performed before the introduction of second harmonics and although eyeballing EF measurement have proven to be robust when performed careful, it is still considered highly subjective and questioned in both clinical and research settings.

The aim of this study was to investigate the correlation and variability of visually assess EF by 2DE and TPE in comparison to quantitative 3DE-EF measurements.

## Methods

### Study population

We included thirty patients who were referred to the echocardiography laboratory at the Department of Cardiology, Karolinska University Hospital, Huddinge. The mean age of the study population was 39 years (range 26 – 66). Table 1 shows baseline characteristics of the included patients.

**Table 1:** Clinical characteristics of the study population.

	**Men**	**Women**
Number (n)	23	7
Age (mean ± SD, years)	40 ± 11	36 ± 16
BSA (m^2^)	2 ± 0.1	1.7 ± 0.1
LV-EF*	55 ± 7	60 ± 4
Sinus rhythm	23	7
HR	66 ± 9	70 ± 13
Significant valvular disease	1	1
DCM	1	0
Diabetes	2	0
Previous AMI	1	0
Hypertonic	1	1
CABG	1	0
PM	1	0
ICD	1	0
Heart failure	2	0
HCM	1	0

* By 3DE, HR: heart rate, DCM: dilated cardiomyopaty, AMI: acute myocardial infarction, CABG: coronary artery bypass grafting, PM: Pacemaker, ICD: implantable cardiovertor defibrillator, HCM: hypertrophic cardiomyopaty.

### Echocardiography

Echocardiographic studies were performed using Vivid 7 Dimension ultrasound equipment, (GE Vingmed Ultrasound, Horten, Norway) by a single experienced operator with patients in a left lateral recumbent position.

Conventional 2DE recordings of standard apical LV 4-and 2 chamber views were acquired using a M4S transducer. Simultaneous TP and RT3DE images were collected using a 3D-array transducer (3V).

**Figure 1:**
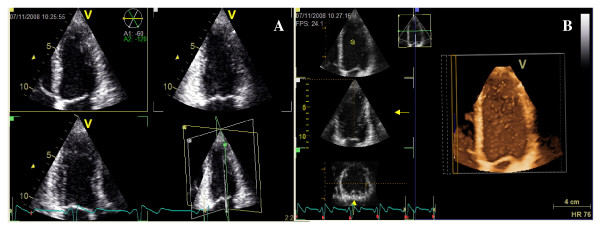
**Triplane echocardiography: 4 – 2 -and 3 chamber views are simultaneously displayed with 4-chamber as the reference view**. Interplane angles are set at 60 degrees **(A)**. A full volume acquisition of left ventricular during four cardiac cycles **(B)**.

When collecting the TP images of the LV, the apical 4-chamber view served as the reference image, and the two other planes were by default displayed with inter-plane angles set to 60 degrees. A figure showing the interposition of imaged planes was simultaneously displayed in a quad screen (figure 1A).

The RT3DE full volume (FV) images (figure 1B) were obtained from an apical window gathered over 4 cardiac cycles during end expiration breath-hold.

The saved 2D, TP and FV cine loops were transferred for a later offline analyze using commercially available software (EchoPAC, GE Vingmed Ultrasound, version 108.0.1).

For the visual 2D-EF evaluation, the 4-and 2 chamber view were displayed simultaneously, and accordingly TP images at different time points in the apical four-two-and three chamber views respectively. Two experienced echocardiographer estimated the 2D and TP LVEF blinded to all clinical data and previous reading. All visual LVEF evaluations were made twice one week apart. The quantitative LVEF evaluations of the RT3D-FV images were made by one reader twice, with one week interval, blinded to the first measurements.

**Figure 2:**
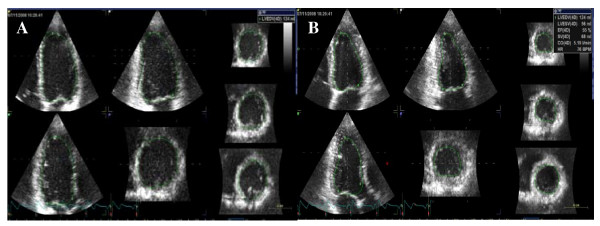
**Three-dimensional echocardiographic ejection fraction measurement**. Automate delineations in diastole **(A) **and systole **(B) **resulting in EF of 55%.

Analyses of the RT3DE data sets were performed using a commercially available semi-automated analysis tool, 4D auto LV volume quantification (4DLVQ, EchoPAC version 108.0.1, GE Vingmed Ultrasound, Horten, Norway). In end-diastole (ED) as well as end-systole (ES) a total of eighteen LV identification landmarks were made: two basal marks at the mitral annulus and one apical mark in the 4-chamber-, 2-chamber- and 3-chamber views respectively (figure 2). Respecting these landmarks, the software automatically delineated the endocardial border in a 3D-model from ED and ES phases. The LVEF is calculated by the software (ED-volume – ES-volume)/ED-volume × 100%. In cases where the automatic delineation of the endocardial border was considered suboptimal the borders could be adjusted manually, although no adjustments were made in this study.

### Statistics

All data are expressed as mean ± SD. Linear regression was performed for correlation analysis. Bland and Altman analysis was used for assessment of agreement between the different methods [17]. In order to determine the intraobserver variability all measurements were analyzed twice with one week apart. The inter- and intraobserver variability was measured according to the following formula: (SD_diff _× 100%)/total mean × √2, where SD_diff _is the SD of difference between measurements. The significance level was set as P < 0.05. Group comparisons of continuous variables were made using analysis of variance (ANOVA). Statistical analysis was performed using standard statistical software (SPSS version 16.0, Inc, Chicago, IL).

## Results

All EF measurements, both 2D and 3D were feasible in all patients. EF was 54.7 ± 8.9% by 3DE, 55 ± 8% by 2D eyeballing and 55 ± 9% by TP eyeballing. Statistical analysis showed no significant differences in EF between the 2DE methods and 3DE.

The mean values and mean differences of EF by the different methods are presented in table 2.

**Table 2:** Ejection fraction assessment by different method.

**Method**	mean ± SD (%)	mean difference (%)

FV3D-EF	54.7 ± 8.9	-0.1 ± 1.8
Eyeballing 2DE	55 ± 8	1.3 ± 2.9
Eyeballing TP	55 ± 9	1.2 ± 2.5

### Regression analysis

**Figure 3:**
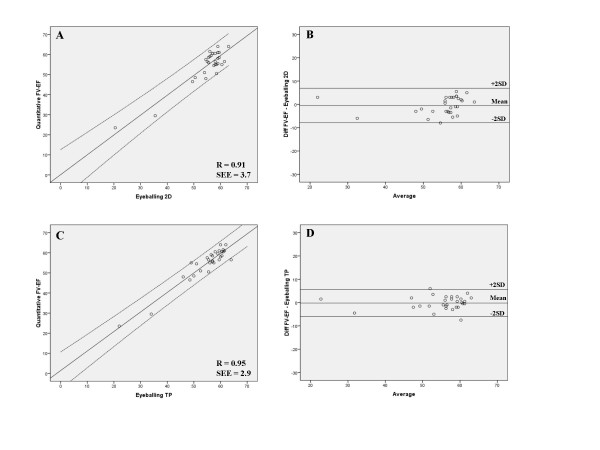
**Linear regression analysis of eyeballing two-dimensional echocardiography EF and quantitative three-dimensional echocardiography EF (*upper left*) and Bland-Altman analysis comparing two-dimensional echocardiography and quantitative three-dimensional echocardiography EF (*upper right*)**. Lines represent regression line (centre) and 95% CI for the mean (outer bounds). Linear regression of eyeballing triplane EF and three-dimensional echocardiography EF (*lower left*) and Bland-Altman analysis comparing eyeballing triplane EF and three-dimensional echocardiography EF (*lower right*).

There were excellent correlations between EF measured by 3D and eyeballing 2DE and TP (r = 0.91 and r = 0.95 respectively). Linear regression between 3D vs eyeballing 2DE and between 3D and eyeballing TP is shown in figure 3.

### Analysis of agreement

The limits of agreement analysis of EF measured by the different methods are shown in table 3. There was a mean difference of -0.5 ± 3.7% for EF determined by 3D and eyeballing 2D and -0.2 ± 2.9% between 3D and eyeballing TP.

**Table 3:** Mean differences and limit of agreement (mean ± SD) for EF determination by the different methods.

	Mean difference ± SD	Limit of agreement	Correlation (r)	SEE
3D-EF minus Eyeballing BP	-0.5 ± 3.7	-7.9 to 6.9	0.91	3.71
3D-EF minus Eyeballing TP	-0.2 ± 2.9	-6 to 5.6	0.95	2.90
Eyeballing BP minus Eyeballing TP	0.3 ± 3.5	-6.7 to 7.3	0.92	3.31

SEE: standard error of estimate.

### Reproducibility of EF

Intraobserver variability was 3.8% for eyeballing 2D, 3.2% for eyeballing TP and 2.3% for quantitative 3D-EF. Interobserver variability was 7.5% for eyeballing 2D and 8.4% for eyeballing TP.

## Discussion

An optimal method for determining LVEF by echocardiography should be rapid, reliable, and widely applicable in order to utilized routinely in a clinical laboratory. Off-line echocardiographic analysis techniques using software's and operator-performed border tracing meet these criteria poorly, explaining their lack of widespread use despite validation in different laboratories [14,15]. The major advantage of visual estimation of LVEF is that the reader can integrate all information regarding wall motion, atrioventricular plane displacement (AV-plane), etc. Its principal limitations are dependency on the skill of the reader and the inter – and intraobserver variability.

In the present study, eyeballing EF both using 2D and TP mode showed excellent correlation with quantitatively measured EF using RT3DE (r = 0.91 and r = 0.95 respectively) and without any significant bias (-0.5 ± 3.7% and -0.2 ± 2.9% respectively). Intraobserver variability was 3.8% for eyeballing 2D, 3.2% for eyeballing TP and 2.3% for quantitative 3D-EF. Interobserver variability was 7.5% for eyeballing 2D and 8.4% for eyeballing TP.

Lavine et al. showed in concordance to our study excellent correlation (r = 0.90) between 2D eyeballing in comparison to radioventriculography (RVG) [18]. In their study they compared eyeballing EF and wall motion scoring (WMS), showing that WMS had better correlation (r = 0.97) in comparison to RVG. This r-value is almost identical to our TP eyeballing determination, where eyeballing has the advantages of being less time-consuming than WMS analysis.

Two advantages of using TPE are that when EF is determined it is based from same cardiac cycle, and that TP can overcome the problem of LV foreshortening. Another advantage is that the acquisition time is reduced because the transducer is not moved to obtain data from multiple views. Although, this advantages didn't transfer into significant differences between 2D and TP-EF determination in our study. There is an apparent trend towards a smaller variability using TP in comparison to 2D. The variability in this study is very small and is likely to be more pronounced in clinical routine. Thus there might be significant advantage in using TP over 2D for EF assessment in everyday clinical routine.

It might be suggested that the accuracy of eyeballing EF is dependent of the skill of the reader and that visual assessment of EF is accurate only in very experienced hands. However, our results are in concordance with those shown by the other groups, indicating that eyeballing EF commonly can be used with a high level of accuracy [12-16]. Indeed Jensen-Urstad et al. showed in their study that Simpson's rule correlated worst (r = 0.45–0.51) when comparing EF by different methods (eyeballing, WMS and AV-plane) in comparison to radionuclide imaging during acute myocardial infarction [13]. Furthermore, it has been shown in a study by Akinboboye et al [19] that eyeballing EF is a method easy to learn. If a person with no previous experience of echocardiography, after each evaluation is given instant feedback from a experienced reader, about 60 cases in joint reading is required to achieve the same accuracy as an experienced echocardiographer.

Even though quantitative measurement of LVEF is generally recommended [20], visual assessments of EF is commonly used in everyday clinical practice due to both suboptimal image quality and lack of time.

The results of the present and prior studies suggest that eyeballing EF could be accepted for clinical use provided that the variability of the eyeballing EF in the echocardiography laboratory is low. Therefore, the variability of using visual estimation of LVEF should be regularly tested in any echocardiography laboratory using this as the recommended method.

### Limitations

The variability in the methods in our study is very low which could limit the generazibility of our data, since a larger variability can be expected in the clinical routine. However we believe that in most laboratories, after adequate training this method could provide reliable estimates of LVEF.

The mean age of the study population was relatively low, and most having normal LVEF. This could partly explain the excellent correlation due to the excepted good image quality. Our findings will therefore have to be confirmed in older populations and also in patients with systolic heart failure.

## Conclusion

Visual estimation of LVEF both using 2D and TP by an experienced reader correlates well with quantitative EF determined by RT3DE. There is an apparent trend towards a smaller variability using TP in comparison to 2D, this was however not statistically significant.

## Competing interests

The authors declare that they have no competing interests.

## Authors' contributions

KS and PG initiated the study. RW and LÅB supervised the study and participated in the interpretation of the results and manuscript preparation. KS and PG performed measurements, made all data conversions, plots and calculations from ultrasound data, and participated in the preparation of the manuscript. KS and AM performed statistical analysis and participated in the interpretation of the results. All authors read and approved the final manuscript.
